# Infrared Small Target Detection Based on Weighted Local Coefficient of Variation Measure

**DOI:** 10.3390/s22093462

**Published:** 2022-05-02

**Authors:** Junmin Rao, Jing Mu, Fanming Li, Shijian Liu

**Affiliations:** 1 Key Laboratory of Infrared System Detection and Imaging Technology, Chinese Academy of Sciences, Shanghai 200083, China; raojunmin@mail.sitp.ac.cn (J.R.); mujing@mail.sitp.ac.cn (J.M.); lifanming@mail.sitp.ac.cn (F.L.); 2 Shanghai Institute of Technical Physics, Chinese Academy of Sciences, Shanghai 200083, China; 3 University of Chinese Academy of Sciences, Beijing 100049, China

**Keywords:** IR small target detection, robust, intricate backgrounds, weighted local coefficient of variation (WLCV)

## Abstract

Robust infrared (IR) small target detection is critical for infrared search and track (IRST) systems and is a challenging task for complicated backgrounds. Current algorithms have poor performance on complex backgrounds, and there is a high false alarm rate or even missed detection. To address this problem, a weighted local coefficient of variation (WLCV) is proposed for IR small target detection. This method consists of three stages. First, the preprocessing stage can enhance the original IR image and extract potential targets. Second, the detection stage consists of a background suppression module (BSM) and a local coefficient of variation (LCV) module. BSM uses a special three-layer window that combines the anisotropy of the target and differences in the grayscale distribution. LCV exploits the discrete statistical properties of the target grayscale. The weighted advantages of the two modules complement each other and greatly improve the effect of small target enhancement and background suppression. Finally, the weighted saliency map is subjected to adaptive threshold segmentation to extract the true target for detection. The experimental results show that the proposed method is more robust to different target sizes and background types than other methods and has a higher detection accuracy.

## 1. Introduction

Robust infrared target detection plays a key role in infrared search and tracking applications, such as space surveillance, remote sensing, object tracking, etc. [1,2]. Due to remote imaging, small IR targets generally occupy fewer pixels in each image and do not have obvious texture features and shape information [3]. When the background is complex, small IR targets can easily be obscured by background clutter (e.g., cloud edges, waves) and noise. Moreover, under the influence of the intrinsic noise of the detector and a poor physical environment, the IR target is blurry, with low contrast and low resolution. Consequently, detecting small IR targets with low signal-to-clutter ratios in various complex scenes is still problematic and difficult.

In recent years, many researchers have presented various IR small target detection algorithms, divided into two categories: single-frame detection and multi-frame detection [4]. The multi-frame detection algorithm has drawbacks such as high computational cost and poor adaptation to the scene. It relies on the correlation between adjacent frames, and the process of scene change can easily lead to the loss of the target. Compared with multi-frame detection algorithms, single-frame detection algorithms are more adaptable to the changes of complex scenes and the mobility of target movements, so their applications are more widespread and have led many researchers to study them.

Typical single-frame detection algorithms generally fall into three distinct categories. The first category is methods based on the spatial consistency of the background, such as top-hat transform [5,6] and max-mean/max-median filtering [7]. These algorithms are all based on the assumption of a consistent spatial background, i.e., that the background is continuous and that the pixels in the local background are highly correlated. These methods generally have good performance with a simple and uniform background, but they are sensitive to noise. For example, mean-pass filtering is more effective at suppressing Gaussian noise but less effective at suppressing impulse noise such as pretzel noise. In contrast, the high-pass filter effectively suppresses the background, but is also susceptible to noise and cannot detect the actual target.

The second category is the low-rank sparse recovery-based method. Gao et al. [8] proposed the Infrared Patch-Image (IPI) model, which assumes that the background and target patches satisfy the low-rank and sparse properties. In this way, detecting IR small targets becomes a recovery problem based on a low-rank and sparse matrix. However, the IPI model uses the L1 norm to describe the sparse metric for small targets, and the low-rank regularization term leads to excessive target shrinkage and noisy residuals. Therefore, many improved algorithms have been proposed, such as weighted infrared patch image (WIPI) [9], reweighted infrared patch-tensor model (RIPT) [10], and so on. These algorithms can detect targets of different sizes effectively. However, they are sensitive to strong edges in the background, so targets with low contrast are easily missed and false alarms are frequently triggered.

The third category is target detection based on the contrast mechanism of the Human Visual System (HVS), which is based on local differences between the target and background. Chen et al. [11] introduced the local contrast measure (LCM) for the problem of target detection in IR images. Subsequent researchers have various proposed improvements for constructing local contrast measures. Han et al. [12]. proposed an improved local contrast measure (ILCM), a ratio-based contrast design. Han et al. used the Gabor kernel to construct the improved difference of Gabor (IDoGb) [13], a difference-based contrast design. The authors in [14] proposed the Multiscale Relative Local Contrast Measure (MRLCM), a ratio-difference-based joint contrast design. These algorithms usually use sliding windows with different sizes to traverse the image to detect targets with different sizes, but the algorithm is not efficient due to the repetitive computation of multiscale operations. Moreover, this multiple scaling can destroy the original size of the target, which is called the “expansion effect” in the literature. To overcome this phenomenon, the Double-Neighborhood Gradient Method (DNGM) [15] has been proposed. The DNGM algorithm uses a fixed-size three-layer sliding window to detect targets of different sizes. It effectively avoids the expansion effect and reduces the complexity of the algorithm due to multiscale operations. However, the algorithm only achieves background suppression, with a high false alarm rate for complex backgrounds. The DLCM algorithm was proposed by Pan et al. [16]. The DLCM algorithm achieves background suppression by computing the diagonal grey-level difference. This algorithm has better background suppression but suffers from the same shortcomings as DNGM. In contrast, the target detection algorithm of variance difference (VARD) [17] proposed by Nasiri et al. has good performance in target enhancement. However, the variance is sensitive to noise since the variance is related to the mean, and targets with low SCR cannot be enhanced effectively, resulting in a high false alarm rate.

This paper proposes a weighted local coefficient of variation (WLCV) method consisting of three stages. At the preprocessing stage, a two-dimensional Wiener filtering is applied to the original image to suppress Gaussian noise and preserve the details of the target. Then, high-pass filtering is performed based on the previously processed image, and the suspicious target regions are obtained. At the detection phase, the system is divided into two modules: the background suppression module (BSM) and the local coefficient of variation (LCV) module. BSM combined with the anisotropy of the target and the difference of gray distribution can effectively suppress most of the background. The background suppression module effectively avoids the expansion effect. LCV introduces the coefficient of variation to the target detection of IR images. It has discrete statistical properties by using the distribution of gray levels of the target and the surrounding background. Unlike discrete statistical properties such as variance and standard difference, the advantage of the coefficient of variation is that it does not require the average value of the reference data and reduces the effect of noise on the average value. The combined advantages of these two modules complement each other, reducing the false alarm rate for complex backgrounds and improving target detection performance. At the target extraction stage, an adaptive threshold operation will be used to extract the true target. Experiments with a large amount of real data show that the algorithm detects small targets in a variety of complex scenes and has a higher detection rate and a lower false alarm rate than existing algorithms.

The organizational framework of this paper is as follows. In Section 2, the proposed method and its various parts are detailed. In Section 3, experimental results are presented. Finally, some concluding remarks are given in Section 4.

## 2. Proposed Methods

**Figure 1:**
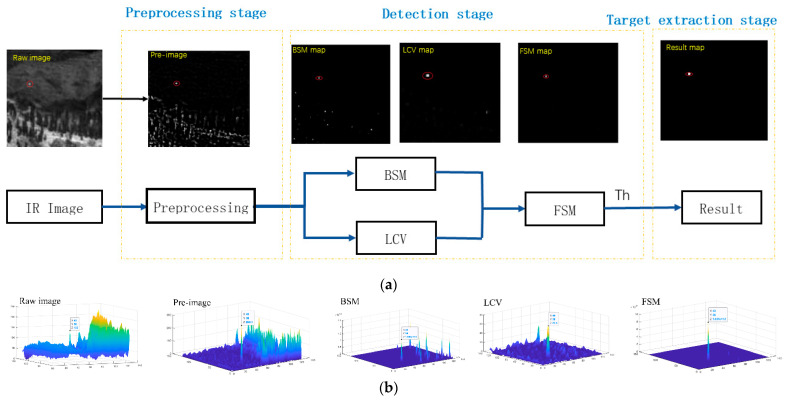
(**a**) The framework of our proposed method. (**b**) Three-dimensional surface after each processing.

The flowchart of the proposed method is shown in Figure 1. The proposed algorithm consists of three important parts. First, the original IR image is preprocessed to extract potential target regions. Second, background suppression and target enhancement are applied to the preprocessed images to obtain saliency maps of the two modules. Finally, an adaptive threshold segmentation mechanism is applied to extract the detected true target from the final weighted saliency map.

### 2.1. Preprocessing Stage

The original IR images are characterized by poor resolution, low contrast, and low signal-to-clutter ratio. If the difference between the gray levels of the target and the background is not obvious, many algorithms directly process the original IR image so that the targets cannot be distinguished from clutter, and even fail to detect targets. Since the spatial distribution of the small targets is approximated by a 2D Gaussian function, the high-pass filter is designed to enhance the targets. However, high-pass filters are sensitive to Gaussian noise. To reduce the effects of noise on target detection and improve the target extraction of the original image, we introduce a preprocessing stage for the original image. At the preprocessing stage, the original IR image is smoothed and denoised to suppress Gaussian noise, improve the details of the target edges, and increase the target extraction capability. Then, the suspect target regions are identified by high-pass filtering based on the previously processed image.

1. Image smoothing and denoising operations are performed to reduce the influence of background clutter on the target detection results. Before high-pass filtering, the image must be able to better preserve the detailed information of the target, such as preserving the edge information of the target and smoothing the noise. The classical methods of image denoising include Wiener filtering algorithms, wavelet algorithms, total variable image denoising algorithms, etc. Wiener filtering can better preserve the detailed features and high-frequency signals of the image, but it can cause blurring effects in the pixel area. The wavelet algorithm can suppress the noise well, but the image details are lost greatly. The total variation algorithm can remove the Gaussian noise and preserve the image details, but it tends to blur the edge details. To preserve the detailed features of the image with the high-frequency signal as much as possible, in this work, a two-dimensional Wiener filter with adaptive characteristics is used to smooth the image for denoising.
2. In the high-pass filtering operation, the smoothed and denoised image is subjected to high-pass filtering. The high-pass filtered image $I_{H}$ is expressed as:

(1) $$I_{H}=I\times G,\;G=\left(\begin{array}{ccc} -1 & -1 & -1\\ -1 & 8 & -1\\ -1 & -1 & -1 \end{array}\right).$$

where I represents the image after the smoothing and denoising operation. As IR small targets generally contain only a few pixels, a common 3 × 3 high-pass filter kernel G is used in this paper.

**Figure 2:**
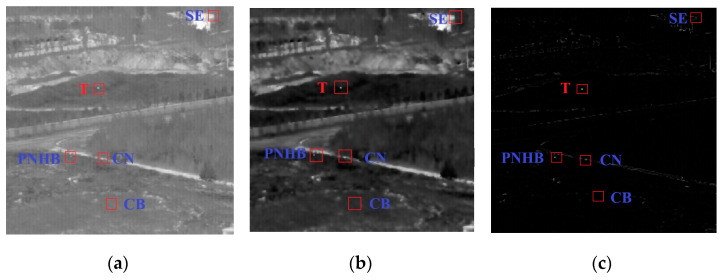
Preprocessing stage map. (**a**) Original IR image. (**b**) Image after being smoothed and denoised. (**c**) High-pass filtering image.

Figure 2a shows a typical sample of a real IR image containing the target (denoted by T), constant background (denoted by CB), clutter noise (denoted by CN), strong edges (denoted by SE), and pixel-sized noise with high brightness (denoted by PNHB). Figure 2b is an image showing the effects of processing the image with the two-dimensional Wiener filter. The edge and high-frequency detail information of the image are better preserved, and the Gaussian noise is suppressed. From Figure 2c, it can be seen that after high-pass filtering, the image is effectively cleared of the low-frequency part of the background and the CB is very cleanly suppressed. However, the CN, SE, and PNHB cannot be filtered effectively. To improve the detection efficiency, this work focuses on two aspects: suppressing complex backgrounds (e.g., CN, SE, PNHB, and other noise) to reduce false alarms, and enhancing targets to improve target detection performance.

### 2.2. Detection Stage

The detection stage consists of two modules, the background suppression module and the local coefficient of the variation enhancement module. The final saliency map (FSM) is created by merging the BSM and LCV. They share the information in the sliding window so that the entire sliding window of the image needs to be traversed only once. The sliding window is from top to bottom and from left to right.

#### 2.2.1. Background Suppression Module (BSM)

The complex background is one of the most important factors affecting the detection performance of the method. In an IR image, there is a grayscale difference between the target and the surrounding background, and the target corresponds to a singularity within a local area with a large grayscale gradient in all directions. The orientation is anisotropic. After the IR image passes through the high-pass filter, the edge features of the background are also enhanced and extracted. The edge features of the background have a strong gradient only in a certain direction and are not anisotropic in all directions like the small target. Therefore, in this work, the small target is extracted based on the difference between the target and the background in terms of grayscale and direction, and the background clutter is further attenuated.

The small target in the IR image often has a small area, less than 9 × 9 according to the Society of Photo-Optical Instrumentation Engineers (SPIE) [11]. Conventional multiscale algorithms, such as LCM, MRLCM, MPCM [18], and DLCM use the maximum local contrast measure in different scales to strengthen the target. When the size of the small target in the IR image is smaller than the sub-window, the multiscale algorithm strengthens the background area around the target, increasing the detected target to the size of the sub-window. We call this the “expansion effect”. The key to solving this effect is to find a non-multiscale method that can adaptively detect targets of different sizes [15].

**Figure 3:**
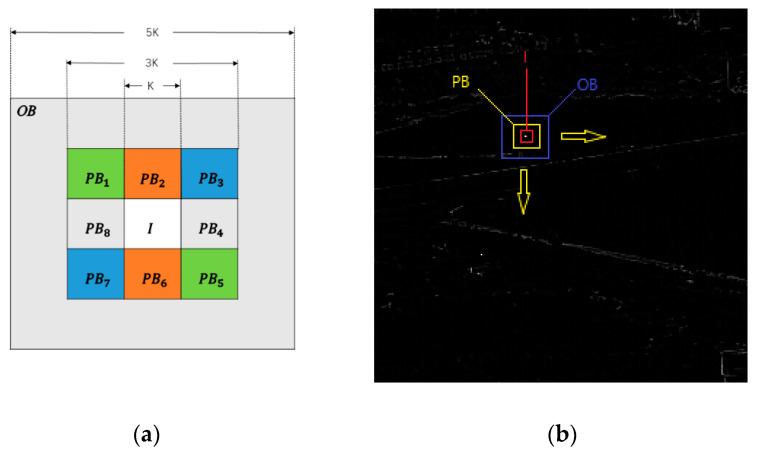
(**a**) The nested structure of the tri-layer window. (**b**) Regions of the tri-layer window.

In order to detect small targets ranging from 2 × 1 to 9 × 9 pixels (a target larger than 80 pixels is no longer considered a small target, which is beyond the scope of this study), a new three-layer window is designed, as shown in Figure 3a. The entire window is divided into three regions, where the innermost region I expresses the reference cell (representing the region where the target might appear). The middle region is protected region $PB_{i}$, which is around *I* with eight sub-windows. The outermost region *OB* expresses the background region. When the target is located in the center of the three-layer window, the differences in orientation and grayscale of the target are reflected in all three regions, regardless of the size of the target, and these differences can be used to detect targets of different sizes.

To extract the background information, the background coefficient (*BC*) is defined using the grayscale difference between the target and the background, which can be defined as follows:

(2) $$BC=H\left[\left(M_{I}-M_{PB})\times (M_{I}-M_{OB}\right)\right]\times H[(M_{I}-M_{PB})+(M_{I}-M_{OB})]$$

(3) $$M=\frac{1}{N}\sum_{n=1}^{N}g_{n}(x,y)$$

(4) $$H(x)=\{\begin{array}{cc} x, & x>0\\ 0, & else \end{array}$$

where, $M_{I}$, $M_{PB}$, and $M_{OB}$ are the mean value of the reference area, the mean value of the protected area, and the mean value of the background area, respectively; (x,y) is the coordinate of each pixel in the region; N is the total number of pixels in a region; $g_{n}$ is the gray value of the nth pixel, and H(·) is the Heaviside step function.

*BC* averages the local grayscale, which can effectively suppress PNHB. However, it cannot effectively suppress clutter with similar grayscale differences to the target. Therefore, this article introduces directional information (*DI* [18,19]) to improve background suppression. The nested structure of Figure 3a shows that the central part *I* is the reference area (targets may appear here), and the protected area is divided into 8 patches $PB_{i}(i=1,2,...8)$. The difference between reference area *I* and protected area $PB_{i}$ is defined as follows:

(5) $$d\left(I,PB_{i}\right)=M_{I}-M_{PB_{i}}\begin{array}{cc} , & \left(i=1,2,...8\right) \end{array}$$

Unlike structured background clutter, small targets have positive contrast in all directions. To characterize this property, the directional measure $\tilde{d_{i}}$ is defined:

(6) $$\tilde{d_{i}}=d\left(I,PB_{i}\right)\times d\left(I,PB_{i+4}\right)\begin{array}{cc} , & \left(i=1,2,\ldots ,4\right) \end{array}$$

where $\tilde{d_{i}}$ measures the difference between the reference area and the peripheral protected sub-windows along the i-th direction. The i-th directions represent the diagonal, vertical, diagonal, and horizontal directions, respectively. When $d\left(I,PB_{i}\right)$ and $d\left(I,PB_{i+4}\right)$ have the same sign, $\tilde{d_{i}}>0$, indicating that the intensity of the reference area is higher (lower) than that of protected area in the i-th direction, and candidate bright (dim) targets may appear in the reference area. On the contrary, when $d\left(I,PB_{i}\right)$ and $d\left(I,PB_{i+4}\right)$ have different signs, $\tilde{d_{i}}<0$, indicating that the reference area is the background area.

As mentioned above, small targets have positive contrast in all directions, while structural background clutter has negative contrast in some directions. Therefore, the minimum value of contrast is chosen to measure the reference area’s directional information (*DI*). Therefore, in this paper, the *DI* is defined by

(7) $$DI=\begin{array}{cc} \underset{i=1,2,\ldots ,4}{\min} & \tilde{d_{i}} \end{array}$$

Based on the previous preparation, the BSM is defined as follows:

(8) BSM=BC×DI

where BSM consists of *BC* weighted by *DI*. *BC* measures the probability that the reference area is background. The closer *BC* is to zero, the more likely it is background. *DI* measures the directional information of the reference area.

**Figure 4:**
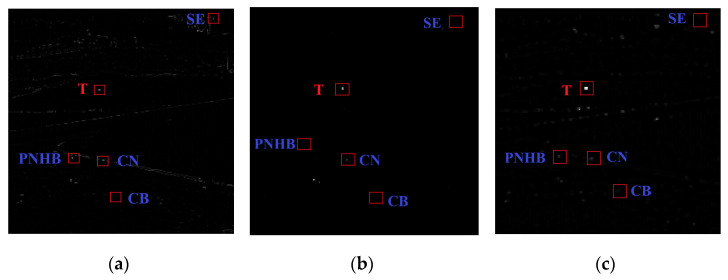
Detection stage maps. (**a**) Preprocessing results map. (**b**) BSM salient map. (**c**) LCV salient map.

From Figure 4b, we will discuss the BSM results for different types of pixels.

1. When the CB appears in the reference area, since the background is usually continuous, we can easily obtain

(9) $$M_{I}\approx M_{PB}\approx M_{OB},BC\approx 0.$$

(10) BC≈0,DI≈0,BSM≈0.

2. When the SE appears in the reference area, since strong edges have directionality, we can easily determine that

(11) BC>=0,DI<0,BSM<=0.

3. When the PNHB, CN, and T appear in the reference area, since PNHB is averaged, both the grayscale difference and the directional gradient difference in the reference area are limited, so we can obtain a BSM that is greater than 0 but has a limited value. However, CN and T are relatively similar in some cases, and after being averaged, it is difficult to distinguish between them simply by the grayscale variability and directionality of the reference area. We can easily get

(12) $$M_{I}>M_{PB},M_{I}>M_{OB},BC>0.$$

(13) BC>0,DI>0,BSM>0.

The BSM value of CN and T is much larger than the BSM value of PNHB. Therefore, it can suppress PNHB noise better.

From the above discussion, we can conclude that after the BSM calculation, T and CN will be the most prominent, and other types of clutter will be suppressed. The next step is to use the enhancement module to distinguish between T and CN.

#### 2.2.2. Local Coefficient of Variation (LCV)

BSM exploits the grayscale variability of different regions while taking into account the orientation properties of the target. BSM is excellent at suppressing complex backgrounds, but its effect on target enhancement is mediocre. To solve this problem, inspired by VARD, the target is enhanced by the difference in the discrete degree of the local grayscale between the target and the background. However, VARD has weaknesses in detection performance and background suppression. Its detection performance depends on the size of the sliding window. In addition, the variance is affected by the mean, and if noise is present, variations in the mean will affect the detection performance. Unlike the characteristic variance of the degree of dispersion, the coefficient of variation can eliminate the influence of the mean. The coefficient of variation removes the effects of inconsistent ranges of grayscale variation in different areas of the image (e.g., background areas and reference areas) and better reflect the degree of dispersion [20].

In statistics, the coefficient of variation (CV) is defined as:

(14) $$\mathrm{CV}=\frac{S}{M}$$

where S is the standard deviation. It is defined by

(15) $$S=\sqrt{\frac{\left(\sum_{n=1}^{N}{\left(g_{n}(x,y)-M\right)}^{2}\right)}{N-1}}$$

Using Equation (16), a local coefficient of variation (LCV) model for the degree of dispersion of the targeted local grayscale is proposed. The proposed model is the following equation.

(16) $$\mathrm{LCV}=\frac{\left(2\times S_{I+PB}-S_{OB}\right)}{M_{OB}+\tau }$$

where $S_{I+PB}$ is the standard deviation of all the grayscale in the protected area and the reference area; $S_{OB}$ is the standard deviation of the grayscale of the background area; and $M_{OB}$ is the mean value of the background area. In statistics, the CV cannot be calculated when the mean of a variable is zero. Therefore, we introduce a hyper-parameter, τ, which is set to be $1\times 10^{-5}$ in this article.

From Figure 4c, the different results of LCV enhancement for different types of pixels are discussed as follows:

(1) When the CB appears in the reference area, since the background is usually continuous, we can easily obtain

(17) $$S_{I+PB}=S_{OB},{\mathrm{LCV}}_{\mathrm{CB}}=\frac{\left(2\times S_{OB}-S_{OB}\right)}{M_{OB}+\tau }=\frac{S_{OB}}{M_{OB}+\tau }.$$

The CB dispersion is small. Therefore, the ${\mathrm{LCV}}_{\mathrm{CB}}$ is also small.

(2) When PNHB and T appear in the reference area, the background means of PNHB and T are similar. The standard deviation refers to the mean value of all the grayscale in the protected area and the reference area. The standard deviations of both PNHB and T are larger than the background standard deviation. Since the target is more discrete than PNHB, the LCV of the target is much larger than that of PNHB. Therefore, we can easily determine that

(18) $$S_{I+PB}>S_{OB},{\mathrm{LCV}}_{T}>{\mathrm{LCV}}_{\mathrm{PNHB}}$$

(3) When SE or CN appear in the reference area, the standard deviations of SE and CN are similar to the target case. However, they have a high probability of bright clutter in the background, which will cause the background mean to be large. Therefore, the LCV of the target is much larger than that of both. We can easily get

(19) $$M_{OB}^{T}<M_{OB}^{\mathrm{SE}},M_{OB}^{T}<M_{OB}^{\mathrm{CN}},{\mathrm{LCV}}_{T}>{\mathrm{LCV}}_{\mathrm{SE}},{\mathrm{LCV}}_{T}>{\mathrm{LCV}}_{\mathrm{CN}}$$

where $M_{OB}^{T}$, $M_{OB}^{\mathrm{SE}}$, and $M_{OB}^{\mathrm{CN}}$ represent the mean value of the background area in the case where the reference area is T, SE, and CN, respectively.

From the above analysis, the LCV enhancement to the target is obvious, but the background suppression aspect is not adequate.

Finally, the FSM is created by weighting the saliency map based on the two modules. It combines the complementary advantages of the two modules to suppress false alarms and improve the detection rate.

(20) FSM=BSM×LCV

**Figure 5:**
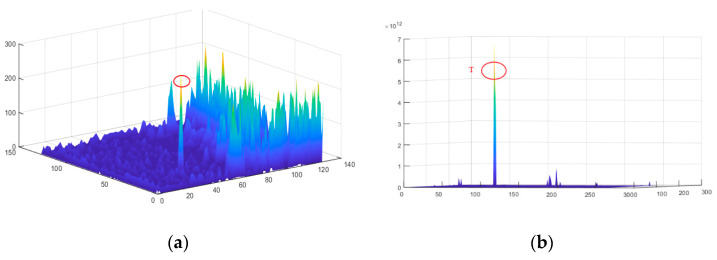
3-D surface. (**a**) 3D diagram of the intensity of the preprocessing results map. (**b**) 3D diagram of the intensity of the FSM map.

Figure 5a,b are the 3D surface of the preprocessing result and the 3D surface of the final saliency map, respectively. With sufficient background attenuation, the objects in Figure 5b can be clearly distinguished.

### 2.3. Target Extraction Stage

From the 3D surface, the target area of the final saliency map is significantly enhanced, and the complex background is sufficiently suppressed. To segment the target from the FSM, an adaptive threshold is used. In this letter, the adaptive threshold Th is denoted by

(21) Th=μ+λ×σ

where μ and σ are the mean value and the standard deviation of the FSM map, respectively. λ is an adjustable parameter, which ranges from 10 to 30.

## 3. Experiment Results

In this section, experiments on real IR datasets to measure the performance of the proposed method in detecting small targets are described. The dataset adopted the single-frame IR small target detection dataset (SIRST) [21]. The SIRST contains a total of 427 images with 480 small targets. The scenes are diverse and include various complex scenes of sea, ground, and air. All experiments were conducted on a laptop with 2.6-GHz Intel Core i7 CPU and 16.0 GB RAM, and the code was implemented in MATLAB 2018b of MathWorks, Massachusetts, USA.

We selected three widely used quantitative evaluation criteria. Quantitative evaluation criteria included signal-to-clutter ratio gain (SCRG), background suppression factor (BSF), receiver operating characteristic (ROC), etc. SCRG was used to evaluate the ability of the algorithm to enhance the target and its expression is:

(22) $$\mathrm{SCRG}=\frac{{\mathrm{SCR}}_{\mathrm{out}}}{{\mathrm{SCR}}_{\mathrm{in}}}$$

where ${\mathrm{SCR}}_{\mathrm{in}}$ and ${\mathrm{SCR}}_{\mathrm{out}}$ represent the signal-to-clutter ratio of the image before and after processing by the algorithm, respectively. SCR is denoted by

(23) $$\mathrm{SCR}=\frac{\left|u_{t}-u_{b}\right|}{\sigma_{b}}$$

where $u_{t}$ is the mean value of the reference region, $u_{b}$ is the average gray of the neighboring background, and $\sigma_{b}$ is the standard deviation of the local background.

The BSF was applied to assess the ability of the algorithm to suppress the background and is expressed as

(24) $$\mathrm{BSF}=\frac{\sigma_{\mathrm{in}}}{\sigma_{\mathrm{out}}}$$

where $\sigma_{\mathrm{in}}$ and $\sigma_{\mathrm{out}}$ are the standard deviations of the background in the image before and after processing by the algorithm, respectively.

The receiver operating characteristic (ROC) reflects the algorithm’s detection performance. ROC describes the relationship between detection probability Pd and the false alarm rate Fa, which is defined as follows [22,23,24]:

(25) $$Pd=\frac{\mathrm{number}\;\mathrm{of}\;\mathrm{true}\;\mathrm{detections}}{\mathrm{number}\;\mathrm{of}\;\mathrm{actual}\;\mathrm{targets}}$$

(26) $$Fa=\frac{\mathrm{number}\;\mathrm{of}\;\mathrm{false}\;\mathrm{detections}}{\mathrm{number}\;\mathrm{of}\;\mathrm{images}}$$

**Figure 6:**
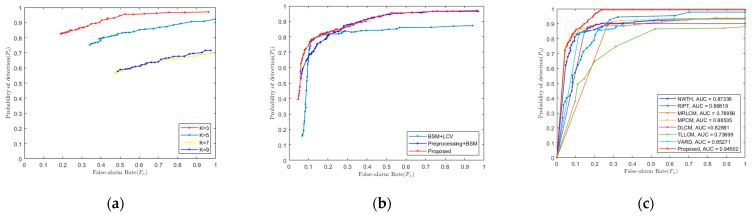
ROC curves. (**a**) ROC curves of SIRST for different K parameters. (**b**) ROC curves of SIRST for different parts of algorithms. (**c**) ROC curves of eight methods in the SIRST dataset.

Because the proposed method uses a three-layer window, it is theoretically possible to detect targets of different sizes for a fixed window size. Parameter *K* is the size of the window cells, which is odd. To test the influence of different *K* parameters on algorithm performance, we used the SIRST dataset. The ROC curves are shown in Figure 6a. It is easy to see that three is the best value. Therefore, in the following experiments, *K* was set to 3.

### 3.1. Ablation Experiment

We performed ablation experiments with different settings to verify the necessity of each part of the algorithm. The ROC curves are shown in Figure 6b. The results of the ROC curves demonstrate that the use of the preprocessing stage greatly improves the performance of small target detection. The proposed method ensures a higher detection rate with a lower false alarm rate than the method without the LCV weighting module.

### 3.2. Comparison to Baseline Methods and Qualitative Analysis

To illustrate the detection performance of the proposed method, it was compared with various types of methods, including the similar algorithms MRLCM [14], MPCM [18], DLCM [16], TLLCM [22], and VARD [17], which are based on the human visual system (HVS) method, New Top-Hat (NWTH) [6], which is based on spatial filtering, and RIPT [10], which is based on sparse representation methods. The detailed parameter settings for each algorithm are given in Table 1. The parameter settings for all compared methods are consistent with the authors’ recommendations.

**Figure 7:**
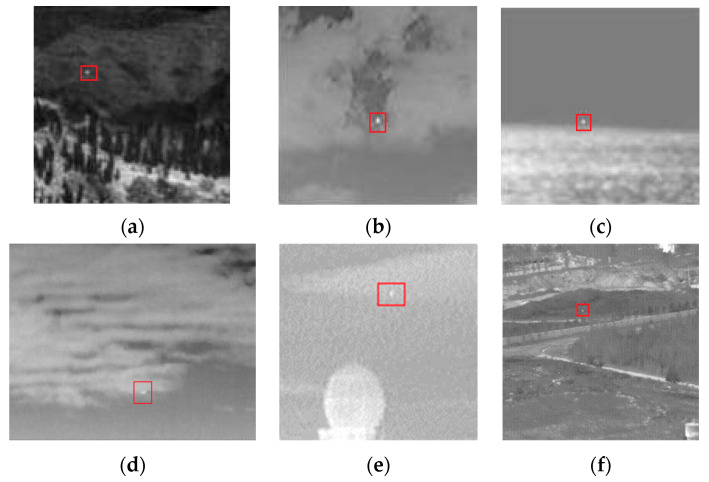
Samples of the six real IR complex scenarios. (**a**–**f**) Scenario 1–Scenario 6.

In this paper, six typical scenarios are selected to qualitatively analyze the performance difference between the proposed algorithm and the comparison algorithm. Figure 7 contains different complex backgrounds, such as sky, ground, and sea, with a variety of clutter types. Scenarios 1 to 5 are from experimental images of different detection algorithms, and scenario 6 is from the dataset [25]. The detailed information of the scenarios is listed in Table 2.

**Figure 8:**
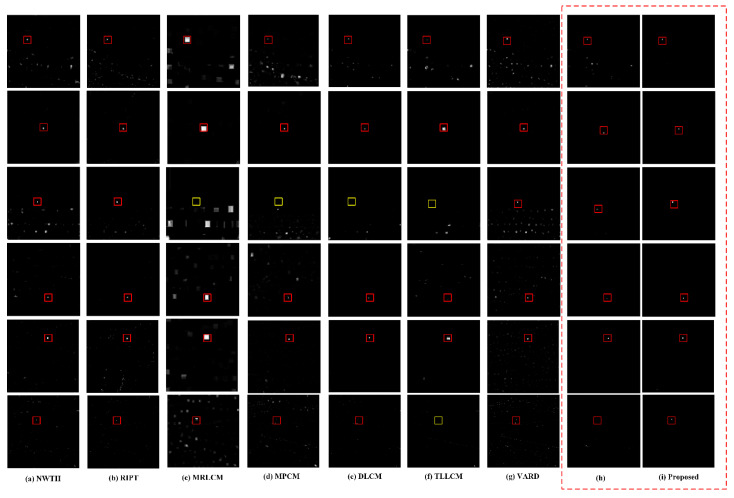
The six real scenes used in qualitative comparison of methods. (**a**) NWTH results. (**b**) RIPT results. (**c**) MRLCM results. (**d**) MPCM results. (**e**) DLCM results. (**f**) TLLCM results. (**g**) VARD results. (**h**) Proposed without preprocessing stage results. (**i**) Proposed results.

Figure 8a–f shows the results of comparison algorithms for detecting small IR targets with different complex backgrounds. Figure 8h represents the result of direct detection of the original image without the preprocessing stage. Figure 8i shows the results of the proposed algorithm. The true targets are marked with rectangles. The red rectangle indicates that the target can be detected, and the yellow rectangle indicates that the target cannot be detected.

The following are the results of the analysis of the proposed algorithm and the comparison algorithm in six scenarios.

1. The NWTH algorithm is unable to suppress strong clutter in the background. There is a high false alarm rate when the background is very complex, as in scenarios 1, 3, 4, 5, and 6.
2. The RIPT algorithm is sensitive to the irregular strong edge and highlight noise, cannot effectively suppress false alarms, and has poor detection performance. There are many false alarms in all scenes and scenario 6 does not effectively distinguish between targets and clutter.
3. The MRLCM algorithm enhances the target but fails to suppress the background, is sensitive to highlight clutter, and has a significant expansion effect. Targets and clutter will be enhanced and will be produced simultaneously, and the target cannot be detected effectively or may even be lost, as in scenario 3.
4. The MPCM algorithm can effectively suppress edges in the background. However, it is sensitive to noise and clutter. Interference noise is amplified and output, as in scenario 1 and scenario 3.
5. The DLCM can only suppress the background but cannot amplify the target. It is sensitive to clutter in complex backgrounds and may result in target misdetection, as in scenario 3.
6. The TLLCM is sensitive to grainy noise in the background and can cause the target to be missed if there is highlight clutter in the background, as in scenario 3 and scenario 6.
7. The VARD algorithm has good performance in target enhancement, but poor background suppression. It enhances the target while amplifying the clutter, which significantly degrades detection performance. Except for scenario 2, VARD caused a large number of false alarms, which severely degraded the detection performance.
8. The proposed algorithm without a preprocessing stage has good detection. However, it is insufficient in suppressing large areas highlighting clutter suppression, as in scenario 1 and scenario 6.
9. The proposed algorithm has excellent background suppression ability and can adapt to various complex backgrounds (e.g., strong edge clutter, point noise, highlight clutter, etc.) and effectively improve the target. The final saliency map has a clean background, and the target is effectively enhanced.

### 3.3. Quantitative Comparisons

Table 3 lists the statistics of SCRG and BSF obtained from the SIRST dataset after processing by different algorithms. SCRG and BSF indicate that the strength of the algorithm can improve targets and suppress complex backgrounds, with larger values being better. Note that the Inf in BSF and SCRG causes the grayscale value of the adjacent background area to be very close to zero after processing. NWTH, MRLCM, MPCM, and TTLCM achieve the goal of simultaneously enhancing the target and suppressing the background. However, the ability to enhance the target and suppress the background is limited. DLCM provides great background suppression but insufficient enhancement of the target. Conversely, VARD is strong in target enhancement but inadequate in background suppression. The RIPT algorithm is a sparsely expressed single-frame detection algorithm, and its calculation results in a target map and a background map, respectively. The SCRG and BSF of the algorithm are calculated from the target map and the value of Inf is obtained, which indicates that the local background region of the target is completely suppressed in the algorithm’s target map. However, RIPT cannot eliminate the interference caused by strong edge clutter. The proposed algorithm takes advantage of the different characteristics of the targets to achieve not only superior background suppression for the BSM module, but also excellent target enhancement for the LCV module.

In this paper, the detection and false alarm rates of each algorithm were calculated on the SIRST dataset, and the ROC curves were plotted, as shown in Figure 6c. The ROC curve of this algorithm is on the top left of the other curves, which means that the detection rate of this algorithm demonstrates the best performance compared to other existing algorithms for the dataset. The AUC is the region enclosed by the ROC curve, which also directly indicates the detection performance. The proposed algorithm also achieves the best results on the dataset.

### 3.4. Discussion

IR small target detection based on contrast mechanism has good prospects for application. The existing algorithms have some drawbacks, such as the expansion effect caused by multi-scale algorithms, and some algorithms are sensitive to window size and noise. This study utilized a non-multiscale three-layer window design to avoid the expansion effect effectively. WLCV fully utilizes the target and various background grayscale characteristics to suppress the complex background while enhancing the target. In addition, to reduce the enhancement effect of clutter, we designed a new measure of LCV weighting, which can better clutter the reduction. The coefficient of variation differs from variance and the standard in that it is more robust to noise and window size. We found that the space-based IR target was characterized by a much stronger clutter intensity than that of the airborne target. This characteristic poses a significant challenge to airborne target detection because existing IR target detection methods enhance the clutter and ignore the real target, leading to missed detections and false alarms [1]. We want to investigate further the detection of small IR targets in different backgrounds in the future.

## 4. Conclusions

In this paper, an effective and robust IR target detection method is proposed. Real targets in complex scenes are easily swamped and disturbed by clutter. Therefore, this method employs preprocessing to improve the robustness of the target in complex scenes. Based on the features of the target and the background, i.e., the local spatial grayscale discrepancy and the gradient direction information, the BSM improves the adaptation to complex backgrounds. Therefore, the target can be detected with a low false alarm rate. The LCV eliminates the influence of the mean fluctuation caused by the size of the sliding window, effectively highlights the target, and reduces false alarms. Experimental results on a dataset containing IR images with complex backgrounds show that the proposed method performs relatively well in both background suppression and detection performance. Compared to other state-of-the-art methods, it has obvious advantages in quantitative parameters such as SCRG and BSF and visual quality.

## Figures and Tables

**Table 1:** Parameter values used in the algorithms.

No.	Methods	Acronyms	Parameter Settings
1	New Top-Hat	NWTH [6]	$S\left(B_{b}\right)=S\left(\Delta B\right)=21,M\left(\Delta B\right)=5$
2	Reweighted Infrared Patch-Tensor Model	RIPT [10]	Patch size: 50 × 50, sliding step 10, $\lambda =\frac{L}{\sqrt{\min\left(I,J,P\right)}},L=1,h=1,\epsilon =0.01,\varepsilon =10^{-7}$
3	Multiscale Relative Local Contrast Measure	MRLCM [14]	$\mathrm{Cell}\;\mathrm{size}:\;9\;\times \;9,\;K_{1}\in \left\{2,5,9\right\},K_{2}\in \left\{4,9,16\right\}$
4	Multiscale Patch-Based Contrast Measure	MPCM [18]	Cell size: 3 × 3, 5 × 5, 7 × 7, 9 × 9
5	Double-Layer Local Contrast Measure	DLCM [16]	N = 3, Local window size: 15 × 15
6	Tri-Layer Local Contrast Measure	TLLCM [22]	c=3,K=9,R∈{5,7,9}
7	Variance Difference	VARD [17]	D = 3, Local window size: 15 × 15
8	Weighted Local Coefficient of Variation	**Proposed**	K=3, Local window size: 15 × 15

**Table 2:** Information of the six scenarios.

Scenario	Resolution	Target Size	Background	Details
1	127 × 127	4 × 3	Mountain-Forest background	Heavy clutter. Strong edge clutter. Random noise.
2	127 × 126	5 × 5	Sky-Cloud background.	Strong edge clutter.
3	127 × 126	2 × 3	Sea-Sky background	Heavy clutter.
4	250 × 200	3 × 4	Sky-Cloud background	Strong edge clutter.
5	127 × 126	3 × 2	Sky	Strong edge clutter. Random noise. Low SCR.
6	256 × 256	2 × 2	Ground	Heavy clutter. Strong edge clutter. Random noise. Bad pixels.

**Table 3:** The statistics of SCRG and BSF of different algorithms.

Data	Evaluation Metrics	NWTH	RIPT	MRLCM	MPCM	DLCM	TTLCM	VARD	Proposed
SIRST	SCRG	4.84	**Inf**	6.42	2.95	0.99	32.42	453.42	**Inf**
	BSF	9.02	**Inf**	13.90	28.02	94.18	38.58	544.97	**Inf**

## Data Availability

Not applicable.
